# Loneliness in Relation to Depression: The Moderating Influence of a Polymorphism of the Brain Derived Neurotrophic Factor Gene on Self-efficacy and Coping Strategies

**DOI:** 10.3389/fpsyg.2017.01224

**Published:** 2017-07-19

**Authors:** Marc Bedard, Robbie Woods, Carly Crump, Hymie Anisman

**Affiliations:** Department of Neuroscience, Carleton University Ottawa, ON, Canada

**Keywords:** depression, polymorphism, single nucleotide, brain derived neurotrophic factor, coping, self-efficacy

## Abstract

Disturbances of brain derived neurotrophic factor (BDNF) signaling, which may occur among those with a polymorphism of the Val66Met gene, comprising a Met substitution for the Val allele, may be associated with depressive cognitions. However, presumed elevated BDNF levels among individuals with the Val/Val genotype, might confer increased responsivity to contextual challenges, thus fostering vulnerability to depression. In Study 1, among undergraduate students (*N* = 252), increased loneliness perceptions were accompanied with depressive symptoms. This relationship was moderated by self-efficacy and BDNF genotype, such that when individuals appraised high self-efficacy, those with the Val/Val genotype, compared to Met carriers, reported greater depression scores when they perceived feeling lonely. Study 2 revealed that among undergraduate students (*N* = 178), lower depressive scores were associated with increased problem-focused coping among Val/Val individuals, but not Met carriers. Moreover, with increased perceived loneliness, Val/Val carriers endorsed lower problem-focused coping. Findings suggest that Val/Val individuals may have adverse neurocognitive vulnerability to loneliness experiences.

## Introduction

Neurotrophins, including brain-derived neurotrophic factor (BDNF), that are fundamental for neuronal plasticity and neurogenesis (Binder and Scharfman, 2004), may be expressed at lower levels following stressor experiences (Duman, 2014). Likewise, there is evidence for a role of BDNF in depressive disorders as serum concentrations of this neurotrophin may be diminished among depressed individuals relative to controls (Shimizu et al., 2003; Wolkowitz et al., 2011; Bus et al., 2015). Moreover, BDNF levels may predict depression severity, as lower BDNF levels may be found among depressed individuals who are suicidal compared to those who are not suicidal (Kim et al., 2007), and relapsed or recurrent-episode patients with major depression have lower BDNF levels than those presenting with a first episode (Lee et al., 2007). Along the same line, antidepressant treatment was linked to decreased depressive symptoms and elevations of BDNF serum levels (Shimizu et al., 2003; Wolkowitz et al., 2011; Bus et al., 2015).

A single nucleotide polymorphism of the BDNF gene, rs6265, comprising a substitution of the amino acid valine (Val) to methionine (Met) at codon 66 in the 5′ pro-region (i.e., Val66Met), may lead to reduced activity-dependent BDNF secretion and trafficking in cortical neurons (Egan et al., 2003; Chen et al., 2004). Perhaps due to lower endogenous BDNF levels, those with the Val66Met polymorphism exhibit greater antidepressant response rates than do depressed individuals homozygous for Val alleles (Zhou et al., 2010). Interestingly, Met carriers may exhibit decreased synaptic plasticity in the hippocampus (Egan et al., 2003) and prefrontal cortex (Liu et al., 2012). Indeed, smaller hippocampal (Frodl et al., 2007; Molendijk et al., 2012), and prefrontal gray-matter volumes (Kim et al., 2013) have been observed, implicating the Val66Met gene in cognitive processes, which may confer greater vulnerability to depression.

The way individuals perceive and cope with stressor experiences has been associated with depressive disorders. For instance, the emergence and maintenance of depressive disorders have been linked to the use of emotion-focused coping, which may comprise emotional containment or expression, blame, withdrawal, passive resignation, or avoidance, rather than the use of problem solving, cognitive restructuring, active distraction, or humor, which coping strategies coinciding with a problem-focused orientation (Matheson and Anisman, 2003; Caldwell et al., 2013). Thus, attention has been devoted to evaluating whether differential use of particular coping styles are linked to specific BDNF genotypes. It was reported that Met carriers may be more likely to endorse emotion-focused coping strategies (Caldwell et al., 2013), such as rumination (Hilt et al., 2007; Beevers et al., 2009), which is often a counterproductive method of coping with stressors (Nolen-Hoeksema, 2000; Joormann et al., 2006) and has been predictive of greater depressive symptomatology (Ravindran et al., 2002; Matheson and Anisman, 2003; Thompson et al., 2010). This is not to say that emotion-focused coping styles necessarily foster depressive symptoms (Stanton et al., 1994), but greater depression can be predicted in certain contexts, for instance, if emotion-focused strategies are used in an inflexible manner (Matheson and Anisman, 2003; Kelly et al., 2007; Nolen-Hoeksema et al., 2008).

Feelings of loneliness have frequently been linked to depressive disorders (Hawkley and Cacioppo, 2010; Cohen-Mansfield et al., 2016), and may exacerbate existent depressive symptoms (Cacioppo et al., 2006). It has been suggested that this relationship may stem from a constellation of persistent depressive cognitions that are tied to perceived social isolation (Cacioppo and Hawkley, 2009), such as emotion-focused coping, which may be employed to a much greater extent and in a more inflexible manner (Revenson, 1980; Schoenmakers et al., 2015). Similarly, disturbed mood might also arise from a lack of social connectivity, which could be fundamental for effective coping (Cruwys et al., 2014).

In addition to the endorsed use of particular coping styles, depressive symptoms may also stem from decreased agency to cope with adverse situations (Luszczynska et al., 2005). Indeed, lower self-efficacy, which refers to perceptions of an inability to cope with stressful or challenging environmental situations (Luszczynska et al., 2005), has been associated with both increased depression scores and feelings of loneliness (Wei et al., 2005; Cohen-Mansfield et al., 2016). Moreover, feelings of loneliness co-occurred with reduced white matter in several brain regions, including the right anterior insula, bilateral inferior parietal lobule, and dorsomedial prefrontal cortex, which are believed to be linked to self-efficacy as well as social cognition (Nakagawa et al., 2015). In effect, depressive symptoms may arise out of an inability to cope with stressors, including feelings of loneliness.

As the Met allele of the Val66Met gene may also be associated with structural and functional changes of prefrontal cortical areas (Liu et al., 2012; Kim et al., 2013), as well as decreased white matter integrity in frontal lobe tracts (Ziegler et al., 2013; Tatham et al., 2016), it might be reasonable to expect that the BDNF genotype would moderate the relations between loneliness, self-efficacy, and depression. This said, because BDNF may serve as a neuroplasticity factor, it could be argued that the influence of BDNF in relation to depressive symptoms could vary with situational influences, as has previously been shown with relations involving serotonin (Belsky et al., 2007, 2009). From this perspective, those presumed to have adequate levels of neuroplasticity may be differentially more sensitive to negative life events (Belsky et al., 2007, 2009). Thus, decreased perceptions of social isolation may have particularly adverse actions on depressive symptoms among individuals with homozygous Val alleles related to the gene for BDNF (Caldwell et al., 2013). As such, it was of interest to assess whether the relationship between loneliness and depression might vary with perceptions of self-efficacy and coping methods used, and if these relationships would be moderated by the Val66Met genotype, as reported in other contexts (Hilt et al., 2007; Beevers et al., 2009; Caldwell et al., 2013). In this regard, it was of interest to determine whether loneliness experiences would be more closely linked to the use of emotion-focused coping styles and poor self-efficacy among Val/Val carriers, presumed to be more sensitive to aversive contexts.

## Materials and methods

### General procedure

Following the provision of written informed consent, participants completed a series of questionnaires to assess current depressive symptoms, and perceptions of social isolation. The present report comprised two studies, which were each part of a larger investigation into gene polymorphisms as predictors of depressive symptomatology. The larger investigation did not involve measures of loneliness amongst all participants. A questionnaire to assess demographic information was administered to all participants, and Study 1 included a measure to assess self-beliefs to cope with difficult situations, whereas Study 2 measured the endorsement of particular coping styles. Upon questionnaire completion, saliva was collected for the analysis of DNA genotyping. Participants were subsequently debriefed and provided with course credit. All procedures were approved by the Carleton University Psychology Research Ethics Board, responsible for reviewing studies involving human participants.

#### Genotyping

Saliva samples for genotyping were collected using Norgen Saliva DNA Isolation Kits (Norgen Biotek Corp., Thorhold, Ontario, Canada). Extraction of the genomic DNA from the sample collection kit was conducted according to the manufacturer's instructions and diluted to approximately equal concentration (20 ng/μL). DNA samples were then genotyped by McGill University and Génome Québec Innovation Centre, Montréal, Canada, where DNA was amplified using multiplex polymerase chain reaction (PCR) followed by template-directed single base extension. One probe per marker was used for template-directed single base extension and the product was desalted using 6 mg of resin. The products were transferred to a 96-well chip by Agena Bioscience nanodispenser, and then were crystalized with a pre-spotted MALDI matrix. The chip was then read by mass spectrometer (MALDI-TOF MS). The primers and probe used in the amplification of the BDNF rs6265 gene during PCR were as follows:

BDNF forward: ACGTTGGATGTACTGAGCATCACCCTGGA
BDNF reverse: ACGTTGGATGGCTTGACATCATTGGCTGAC
BDNF probe: TCCAACAGCTCTTCTATCA

## Study 1

### Participants

Participants consisted of 252 White/Euro-Caucasian Carleton University undergraduate male (*n* = 73) and female (*n* = 179) students with a mean age of 20.14 (*SD* = 4.87), who were recruited through the university's online computerized recruitment system. A majority were living with friends or roommates on (*n* = 75) or off-campus (*n* = 36), or were living with parents (*n* = 87), with the rest reporting living alone (*n* = 25), living with a significant other (*n* = 13), and 16 specified other arrangements (e.g., living with another relative or with children).

### Measures

#### Depressive symptoms

The Beck Depression Inventory (BDI; Beck et al., 1961) was used to evaluate symptoms of depression. The BDI consists of 21 items corresponding to different depressive symptoms, and for each item, participants selected one of four options, which ranged from low to high symptomatology. Total scores were calculated by summing across all items (α = 0.92), with higher scores indicating greater depressive symptomatology.

#### Loneliness

Perceptions of loneliness and feelings of social isolation were measured using the UCLA Loneliness Scale (Version 3; Russell, 1996). This 20-item scale consists of a list of statements, such as “How often do you feel isolated from others?” or “How often do you feel close to people?” Participants respond on a 4-point likert scale ranging from 1 (never) to 4 (Always), to indicate how often they felt as described by each of the items. Scores were summed to create a total score (α = 0.95), such that higher scores indicate more perceived loneliness.

#### General self-efficacy

The 10-item General Self-Efficacy Scale (Schwarzer and Jerusalem, 1995) was used to assess self-beliefs to cope with difficult situations. Participants responded to items (e.g., “If I am in trouble, I can usually think of a solution”) on a 4-point likert scale ranging from 1 (not at all true) to 4 (exactly true). Total scores were acquired by summing all items (α = 0.89), with higher scores indicating greater degrees of self-efficacy.

### Statistical analyses

Statistical analyses were performed using SPSS version 21 (Armonk, NY, USA: IBM Corp.). All continuous variables were checked for normality and were found to be acceptable with the use of Q-Q plots, and through assessing skew statistics, which remained between −2 and 2 (Curran et al., 1996). Independent *t*-tests were performed to assess differences between scores of depression, self-efficacy, and loneliness as a function of BDNF genotype. Pearson correlations were used to evaluate relations between perceived self-efficacy, depressive symptoms, and loneliness, and relations among categorical variables were analyzed with chi-squared tests. Moderations were analyzed using hierarchical linear regressions, and significant moderations were followed up using a web utility for simple slopes (Preacher et al., 2006). Moderation analyses were conducted using bootstrapping procedures to determine 95% confidence intervals based on 5,000 resamples. Standardized scores were used for all regression analyses. The significance level was set at *p* < 0.05 for all analyses.

## Results

There were six participants for whom genotype could not be determined based on the samples provided, so they were excluded from analyses. Allele distribution for the Val66Met polymorphism, rs6265, consisted of 5 Met/Met (4 female, 1 male), 71 Val/Met (47 female, 24 male), and 176 Val/Val (128 female, 48 male), which met Hardy-Weinberg Equilibrium expectations, $\chi_{\left(2\right)}^{2}$ = 0.49*, p* = 0.780. As a result of the infrequency of the Met/Met allele variant, data from both Val/Met and Met/Met were collapsed together for analyses, as is the convention from prior studies (Caldwell et al., 2013). Genotype distributions were subsequently not found to differ as a function of gender, $\chi_{\left(1\right)}^{2}$ = 0.82*, p* = 0.367.

Moreover, depression scores were not found to differ between Val/Val (*M* = 10.96, *SE* = 0.75) and the composite Val/Met and Met/Met group (*M* = 10.22, *SE* = 1.09), *t*_(250)_ = 0.55, *p* = 0.583, 95% CI = [−1.91, 3.394], *d* = 0.08. Similarly, loneliness did not differ between Val/Val individuals (*M* = 42.18, *SE* = 0.94) and the Met carriers (*M* = 42.38, *SE* = 1.27), *t*_(250)_ = −0.12, *p* = 0.904, 95% CI = [−3.46, 3.06], *d* = 0.02. Those with the Val/Val genotype (*M* = 30.24, *SE* = 0.42) were also not found to differ from Met carriers (*M* = 29.92, *SE* = 0.49) with respect to general self-efficacy, *t*_(250)_ = 0.45, *p* = 0.653, 95% CI = [−1.08, 1.72], *d* = 0.06.

**Figure 1:**
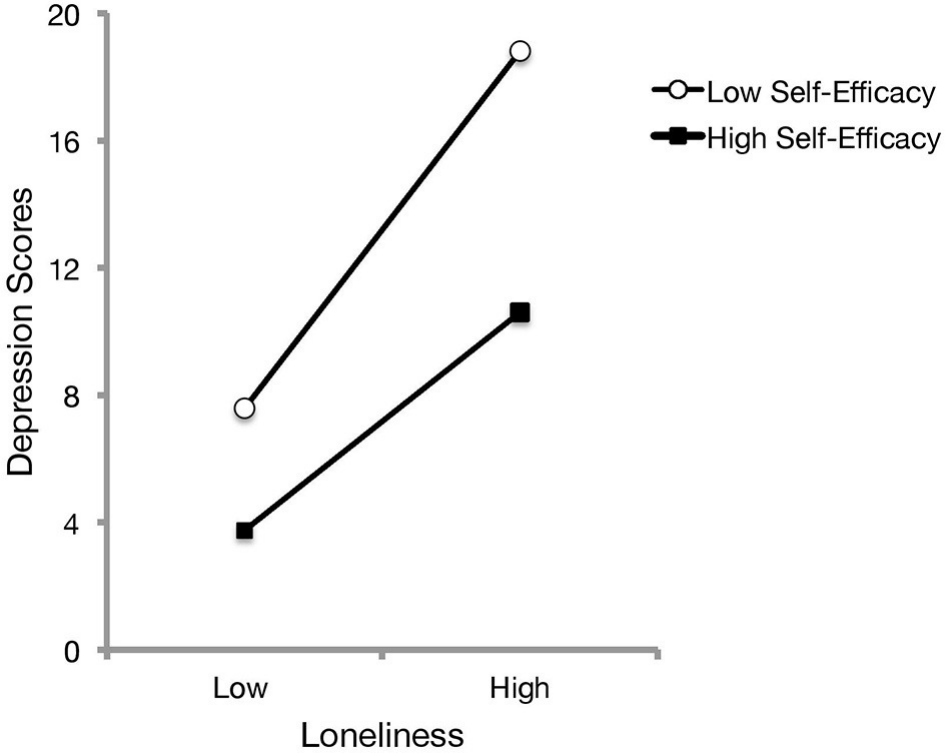
The relation between feelings of loneliness and depression scores as a function of self-efficacy beliefs. Based on hierarchical linear regressions, increased perceived loneliness was associated with elevated depressive symptoms. Plotted separately as a function of low (1 SD below the mean) and high scores (1 SD above the mean) of self-efficacy beliefs, this relationship was more pronounced for those who appraised low self-efficacy, particularly at elevated feelings of loneliness.

Examination of bivariate relationships revealed that loneliness was positively associated with depressive symptoms, *r* = 0.63, *p* < 0.001, and negatively with self-efficacy scores, *r* = −0.51, *p* < 0.001. In addition, general self-efficacy was negatively related to depression scores, *r* = −0.58, *p* < 0.001. Thus, it was of interest to examine whether self-efficacy moderated the relationship between perceived loneliness and depressive symptomatology. To this end, a hierarchical linear regression was conducted with loneliness and self-efficacy in the first step, followed with the loneliness x self-efficacy interaction term added in the second step. These analyses revealed that self-efficacy significantly moderated the relationship between loneliness and depression, Δ*R*^2^ = 0.02, *b* = −1.10, *t* = −2.82, *p* = 0.005. As presented in Figure 1, follow-up simple slope analyses (Preacher et al., 2006) indicated that although increased loneliness was associated with greater depressive scores for both low and high perceived self-efficacy, individuals with increased perceptions of social isolation and that appraised a low ability to cope with difficult situations exhibited more pronounced depressive symptomatology [*b* = 5.62, *SE* = 0.66, *t*_(248)_ = 8.57, *p* < 0.001] than those who appraised a high ability to cope with difficult situations [*b* = 3.43, *SE* = 0.63, *t*_(248)_ = 5.47, *p* < 0.001]. The relationship remained unchanged when age, gender and living arrangement were entered as covariates.

**Figure 2:**
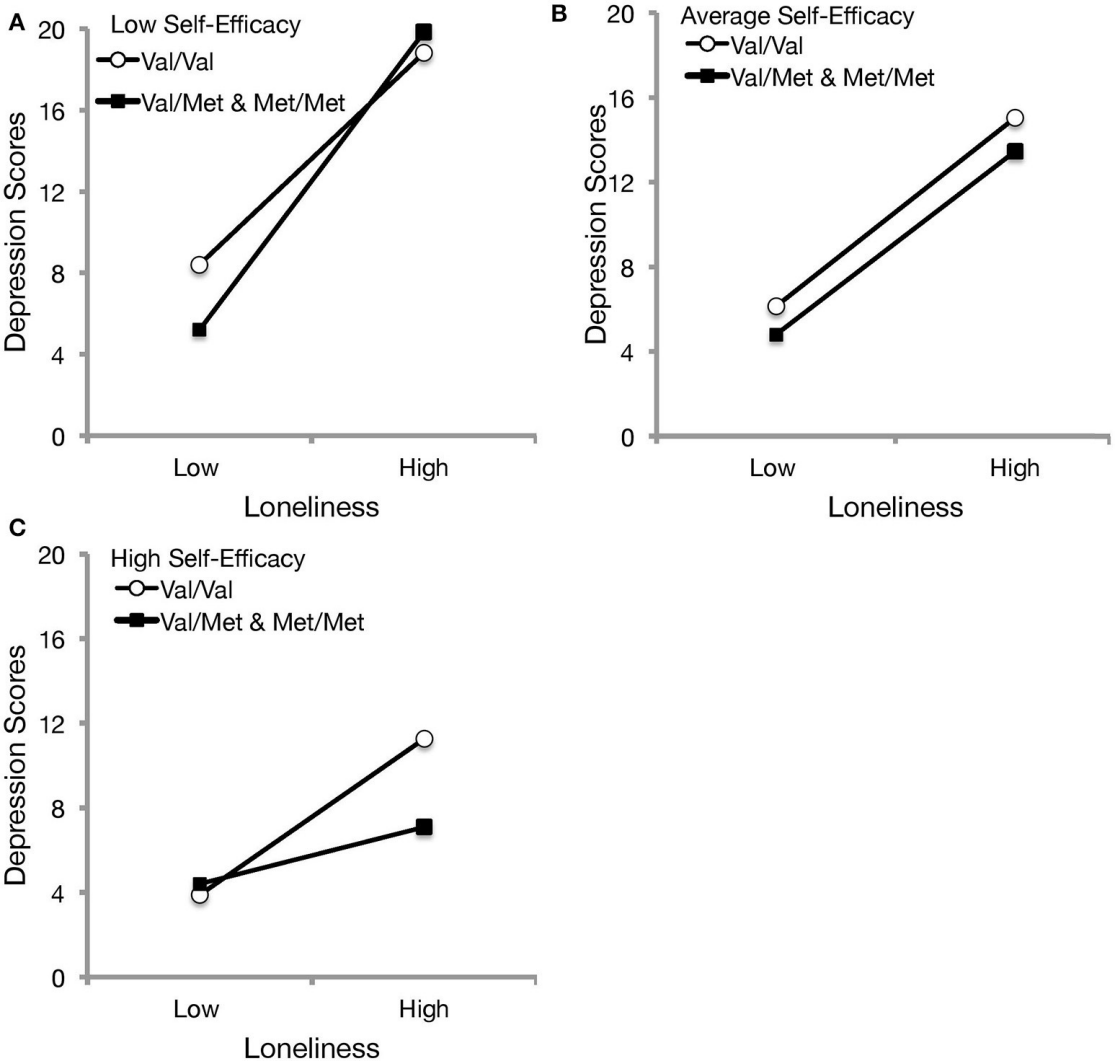
The moderational influences of BDNF genotype and self-efficacy beliefs on the relations between loneliness and depression. At 1 SD below the mean of self-efficacy **(A)**, depressive scores were more pronounced with increased loneliness experiences, particularly among Met carriers. At mean levels of self-efficacy **(B)**, depressive symptoms were similar, though slightly elevated among Val/Val individuals across loneliness perceptions. At 1 SD above the mean of self-efficacy **(C)**, Met carriers exhibited consistent depression scores across levels of social isolation, whereas those with homozygous Val alleles reported elevated depressive symptoms with increased loneliness perceptions.

As it was of interest to examine whether depression would differ on levels of loneliness and self-efficacy as function of BDNF genotype, a double-moderation was performed. Again, hierarchical regression was performed in which loneliness, self-efficacy, and BDNF were entered in the first step. In the second step, interaction terms for loneliness x self-efficacy, loneliness × BDNF, and self-efficacy x BDNF were entered, followed by the loneliness x self-efficacy × BDNF in the third step. Analyses revealed a significant double moderation (Figure 2), Δ*R*^2^ = 0.01, *b* = −2.23, *t* = −1.98, *p* = 0.049, such that lower levels of self-efficacy were associated with an amplification of depressive scores among those who perceived greater loneliness experiences; however, simple slopes analyses with Bonferroni correction indicated that although Val/Val individuals had elevated depression scores at lower perceived loneliness if they appraised low self-efficacy [*b* = 5.52, *SE* = 0.78, *t*_(248)_ = 6.61, *p* < 0.001], those that had a Met allele exhibited the most pronounced depressive symptoms with high loneliness experiences [*b* = 7.32, *SE* = 1.30, *t*_(248)_ = 5.62, *p* < 0.001]. At mean levels of general self-efficacy, Val/Val individuals [*b* = 4.45, *SE* = 0.60, *t*_(248)_ = 7.36, *p* < 0.001] and Met carriers [*b* = 4.33, *SE* = 0.97, *t*_(248)_ = 4.48, *p* < 0.001] exhibited similar levels of depressive symptoms in the presence of loneliness experiences, but interestingly, Met carriers who appraised high levels in their ability to cope no longer exhibited a significant relationship between loneliness and depressive symptoms [*b* = 1.34, *SE* = 1.53, *t*_(248)_ = 0.88, *p* = 0.382], whereas those with the homozygous Val allele exhibited a marked increase in depression scores [*b* = 3.69, *SE* = 0.70, *t*_(248)_ = 5.27, *p* < 0.001]. Furthermore, the findings from the double moderation did not differ when controlling for age, gender, and living arrangement.

## Study 2

It has been reported that compared to individuals homozygous for the Val allele, Met carriers exhibited greater proclivity to use emotion-focused coping strategies (Hilt et al., 2007; Beevers et al., 2009; Caldwell et al., 2013), and the use of emotion-focused coping was typically accompanied by more pronounced depressive symptoms (Ravindran et al., 2002; Matheson and Anisman, 2003; Thompson et al., 2010). However, the moderating role of the BDNF genotype on relationships between coping styles and depressive symptoms (when these variables are included in the same analyses) has not previously been examined.

In addition, Study 1 indicated that loneliness experiences may be associated with lower self-efficacy to cope with difficult situations. Moreover, there was a greater effect of loneliness on depression scores in Val homozygotes in the context of high self-efficacy, whereas Met carriers were more sensitive to increased loneliness when they perceived low coping self-efficacy. It was therefore of additional interest in Study 2 to examine whether those with Val/Val alleles exhibit similar sensitivity to perceived loneliness compared to Met carriers, and report increased use of emotion-focused and decreased use of problem-focused coping with increased loneliness.

### Participants

Participants comprised of 178 White/Euro-Caucasian undergraduate male (*n* = 50) and female (*n* = 128) students with a mean age of 19.91 (*SD* = 5.62), who were recruited through the University's online computerized recruitment system. At the time of assessment, a majority were living with friends or roommates on (*n* = 59) or off-campus (*n* = 28), or were living with parents (*n* = 58), with the rest reporting living alone (*n* = 17), living with a significant other (*n* = 6), and 10 specified other arrangements (e.g., living with a relative or with children).

### Measures

In Study 2 participants completed the BDI (α = 0.89), and the UCLA Loneliness Scale (α = 0.94), as described in the preceding study. In addition, proclivity to use particular coping strategies as a means to deal with problems or recent stressors was assessed through the 50-item Survey of Coping Profile Endorsed (Matheson and Anisman, 2003), with responses recorded on a 5-point likert scale from 0 (never) to 4 (almost always). A principal components analysis with varimax rotation was conducted to determine the underlying factor structure, with items being included on a factor when factor loadings were greater than 0.32, as recommended by Tabachnick and Fidell (2013). This revealed a two-factor solution, which encompassed emotion- and problem-focused coping. Emotion-focused coping consisted of wishful thinking, passive resignation, avoidance, emotional containment, rumination, emotional expression, blaming others, and self-blame (α = 0.82). Problem-focused coping comprised of problem solving, cognitive restructuring, active distraction, and humor (α = 0.69).

### Statistical analyses

Statistical analyses were performed using SPSS version 21 (Armonk, NY, USA: IBM Corp.). As in Study 1, Normality of continuous variables was checked and found to be acceptable with the use of Q-Q plots, and with skew statistics, which remained between −2 and 2 (Curran et al., 1996). Independent *t*-tests were performed to assess differences between scores of depression, problem-focused and emotion-focused coping, and loneliness as a function of BDNF genotype. Pearson correlations were used to evaluate relations between problem-focused coping, emotion-focused coping, depressive symptoms, and loneliness, and relations among categorical variables were analyzed with chi-squared tests. Moderations were analyzed using hierarchical linear regressions, and the significant moderations were followed up using a web utility for simple slope analyses (Preacher et al., 2006). The significance level was set at *p* < 0.05 for all analyses.

## Results

Allelic distribution for the Val66Met polymorphism consisted of 4 Met/Met (3 female, 1 male), 41 Val/Met (28 female, 13 male), and 133 Val/Val (97 female, 36 male), which met Hardy-Weinberg Equilibrium expectations, $\chi_{\left(2\right)}^{2}$ = 0.49*, p* = 0.924. Subsequently, as in Study 1, data from both Val/Met and Met/Met were pooled together for analyses. Genotype distributions did not differ as a function of gender, $\chi_{\left(1\right)}^{2}$ = 0.27, *p* = 0.60.

As with Study 1, no differences were observed between Val/Val (*M* = 8.75, *SD* = 7.66) and Val/Met and Met/Met groups (*M* = 9.84, *SD* = 7.21), on depression scores *t*_(176)_ = −0.84, *p* = 0.404, 95% CI = [−3.67, 1.48], *d* = 0.15, or on perceived loneliness (Val/Val, *M* = 41.56, *SD* = 11.29; Met carriers, *M* = 42.67, *SD* = 11.51), *t*_(176)_ = −0.56, *p* = 0.574, 95% CI = [−4.96, 2.76], *d* = 0.10. Similarly, upon evaluation of coping styles, Val/Val individuals (*M* = 1.95, *SD* = 0.62) did not differ from Met carriers in terms of the use of emotion-focused coping, *t*_(250)_ = 0.276, *p* = 0.783, 95% CI = [−0.18, 0.24], *d* = 0.05, nor did Val/Val individuals (*M* = 2.40, *SD* = 0.65) differ from Met carriers (*M* = 2.28, *SD* = 0.54) on problem-focused coping, *t*_(176)_ = 1.12, *p* = 0.265, 95% CI = [−0.09, 0.33], *d* = 0.20.

An assessment of Pearson product moment coefficients between study variables, once again revealed that loneliness was positively associated with depressive symptoms, *r* = 0.63, *p* < 0.001. As expected, a pattern emerged such that each of loneliness (*r* = 0.55, *p* < 0.001) and depressive symptoms (*r* = 0.61, *p* < 0.01) were positively associated with emotion-focused coping, and negatively with the use of problem-focused coping, (*r* = −0.21, *p* = 0.006, and *r* = −0.21, *p* = 0.006, respectively).

**Figure 3:**
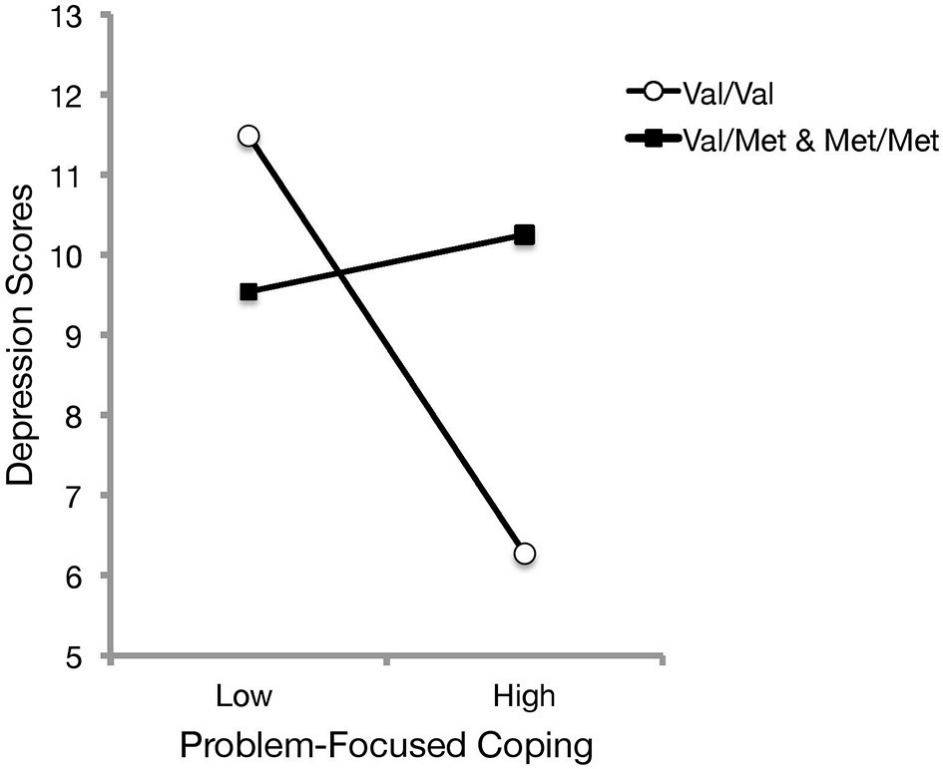
The relation between problem-focused coping and depression scores was moderated by BDNF genotype. Simple slopes analyses indicated that Met carriers exhibited consistent levels of depressive symptoms regardless of the use of problem-focused coping. However, depressive symptoms decreased among Val/Val carriers when more problem-focused coping was reported.

As it was of interest to investigate whether an interaction existed between BDNF genotype and coping styles in relation to depressive symptoms, a hierarchical linear regression was conducted with problem-focused coping and BDNF in the first step, followed with the BDNF genotype x problem-focused coping interaction in the second step. The analysis revealed that BDNF genotype significantly moderated the relationship between problem-focused coping and depression, Δ*R*^2^ = 0.02, *b* = 2.96, *t*_(174)_ = 2.12, *p* = 0.036. As presented in Figure 3, follow-up simple slope analyses (Preacher et al., 2006) indicated that it was only for those with homozygous Val alleles that greater use of problem-focused coping was associated with lower depressive scores [*b* = −2.61, *SE* = 0.61, *t*_(174)_ = −4.30, *p* < 0.001], whereas those who carried at least one Met allele exhibited consistent levels of depressive symptoms regardless of differing use of problem-focused coping [*b* = 0.36, *SE* = 1.26, *t*_(174)_ = 0.28, *p* = 0.778]. An examination into the interaction between BDNF genotype and emotion-focused coping on depressive scores was not significant, [*b* = −0.42, *t*_(174)_ = −0.41, *p* = 0.686]. Neither of these relations were found to change when controlling for age, gender, and living arrangement.

**Figure 4:**
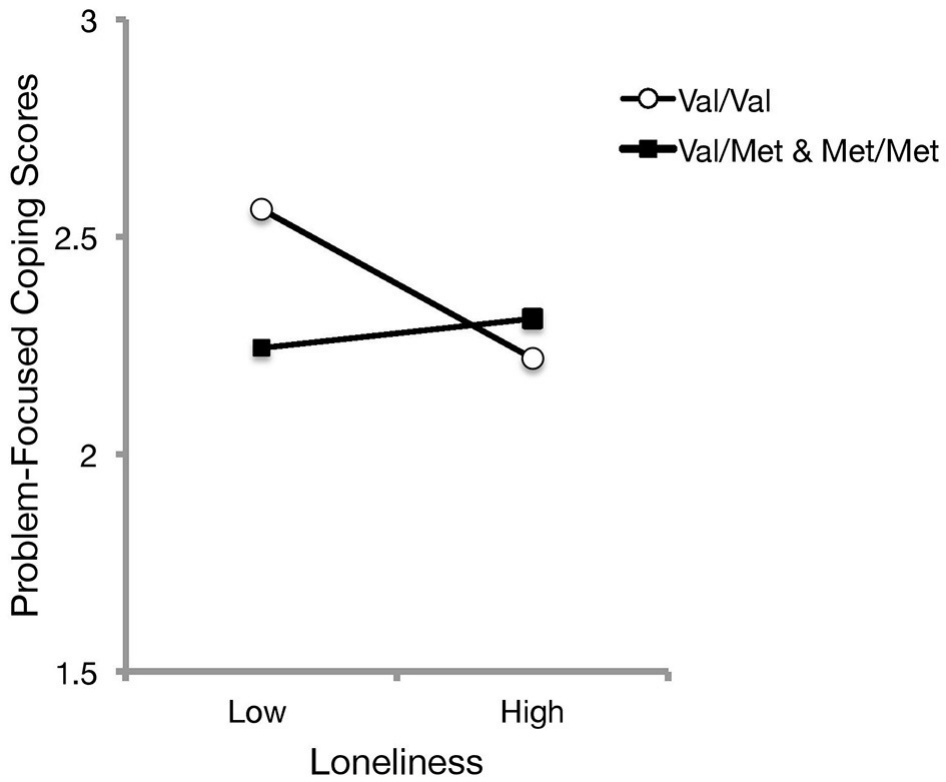
The relation between perceived loneliness and depression scores as a function of BDNF genotype. Simple slopes analyses indicated that Met carriers did not differ in the use of problem-focused coping with increased loneliness experiences. However, problem-focused coping was endorsed to a greater extent with decreasing levels of perceived loneliness among those with the Val/Val genotype.

To evaluate whether coping styles, as predicted by loneliness experiences, differed as a function of the BDNF genotype, further hierarchical linear regressions were conducted with perceived loneliness in the first step, and the loneliness x coping style (i.e., emotion-focused and problem-focused, in separate respective analyses) interaction terms were added in the second step. These analyses indicated that the BDNF genotype did not significantly moderate the relation between loneliness and emotion-focused coping [*b* = 0.03, *t*_(174)_ = 0.35, *p* = 0.725], but a significant interaction was evident between BDNF genotype and loneliness in relation to problem-focused coping scores, Δ*R*^2^ = 0.03, *b* = 0.24, *t*_(174)_ = 2.28, *p* = 0.024. Follow-up simple slope analyses (Figure 4), indicated that use of problem-focused coping was greater at lower levels of perceived loneliness among those with the Val/Val genotype, and that with increasing perceptions of loneliness, the use of problem-focused coping was reduced [*b* = −0.56, *SE* = 0.21, *t*_(174)_ = −2.61, *p* = 0.01]. Among Met carriers, the use of problem-focused coping did not differ with increased loneliness experiences [*b* = 0.40, *SE* = 0.36, *t*_(174)_ = 1.11, *p* = 0.270]. These findings did not differ when age, gender, and living arrangement were entered as covariates.

## Discussion

Both depression and the Met allele of the Val66Met polymorphism on the gene coding for BDNF may be accompanied by reductions in structural and functional connectivity of the hippocampus and prefrontal cortex (Frodl et al., 2007; Molendijk et al., 2012; Kim et al., 2013; Ziegler et al., 2013; Tatham et al., 2016), and BDNF concentrations in serum have been linked to depressive disorders (Shimizu et al., 2003; Wolkowitz et al., 2011; Bus et al., 2015). However, as evidence for a direct relationship between BDNF genotype and depression has not been consistently observed (Verhagen et al., 2010), it was of interest to examine whether the Met polymorphism on the BDNF gene would be associated with cognitive processes that might confer greater vulnerability to depressive symptoms.

### Interactions with self-efficacy appraisals

It is worth considering that certain stressors, including loneliness experiences, may co-occur with or give rise to depressive symptoms (Cacioppo et al., 2006; Hawkley and Cacioppo, 2010; Cohen-Mansfield et al., 2016), which may also be engendered through decreased self-efficacy to cope with stressful situations (Wei et al., 2005; Nakagawa et al., 2015; Cohen-Mansfield et al., 2016). Results from Study 1 (Figure 1) corroborated these previous investigations, as individuals who reported more social isolation exhibited elevated depressive symptoms if they also felt that they lacked self-efficacy in their ability to cope with difficult situations. Interestingly, the BDNF rs6265 genotype moderated the relationship between depression, self-efficacy and loneliness (see Figure 2). When individuals perceived elevated self-efficacy, homozygous Val individuals exhibited more pronounced depressive symptoms at high levels of loneliness, whereas those with a Met allele did not demonstrate a significant loneliness-depression relationship. Evidently, the relationship between disturbances in neurotrophic signaling and coping processes may not be as straightforward as previously thought. In effect, the influence of the Val66Met gene coding for BDNF seems to interact with psychological factors related to stressful experiences and feelings of self-efficacy to cope with difficult situations.

There is evidence that reduced BDNF secretion (Egan et al., 2003; Chen et al., 2004), as well as altered functional and structural connectivity of prefrontal cortical areas (Liu et al., 2012; Kim et al., 2013) may be linked to Met carriers, which may presumably render greater neurocognitive vulnerability to depression. However, findings from the present investigation seem contrary to that notion, and run against the prevailing view that the greater availability of BDNF among homozygous Val carriers may provide (although inconsistently noted) neuroprotection against depressive symptoms (Verhagen et al., 2010), as Study 1 did not reveal group differences on depression scores as a function of BDNF genotype. The present findings suggest that this relationship may be more nuanced, aligning instead with the perspective that genes allowing for increased neurotrophic support and thus enhanced neuroplasticity, would be associated with strengthening of responses to both positive and negative stimuli (Belsky et al., 2007, 2009). As such, the presence of the Val/Val genotype, and hence the presence of sufficient BDNF levels and greater neuroplasticity, may dispose individuals to depressive symptoms in the context of perceiving greater loneliness, despite the presence of high self-efficacy appraisals (Figure 2C). In essence, these findings from Study 1 are consistent with the notion that relative to Met carriers, individuals with the Val/Val genotype may be more sensitive to adverse life experiences including loneliness, and thus are more apt to report depressive symptomatology (Caldwell et al., 2013).

### Influences on coping strategies

However, the actions of the Val66Met gene on relations with depression may not simply be isolated to an appraised ability to cope with difficult situations, and they may also extend to the endorsed use of particular coping strategies. As was mentioned earlier, previous studies had indicated that Met carriers might be more likely to endorse emotion-focused styles (Hilt et al., 2007; Beevers et al., 2009; Caldwell et al., 2013), which had been presumed to be due to altered neuroplasticity. The findings from Study 2, at least initially, were inconsistent with these earlier data concerning the relationship between neurotrophic disturbances and the use of specific coping processes, as homozygous Val carriers were not found to differ from Met carriers in relation to the use of either emotion- or problem-focused coping. However, the data pointed to those with the homozygous Val genotype being more likely to benefit from problem-focused coping strategies, as they exhibited less affective dysfunction (Figure 3). Given that those with homozygous Val alleles did not differ from Met carriers on depressive symptomatology, these data support the position that genes coding for neurotrophins are aligned with enhanced proclivity to adopt problem-focused coping, which may buffer against depression (Ravindran et al., 2002; Matheson and Anisman, 2003; Thompson et al., 2010).

Another stated aim of Study 2 was to separately investigate actions of the Val66Met gene on relations between loneliness and particular coping styles. There is mounting evidence supporting a role of neurotrophins in appraisals and coping in association with stressor experiences (Beevers et al., 2009; Caldwell et al., 2013). It had been reported that emotion-focused coping, which was often an infective coping strategy to deal with stressors, might be more apparent among Met carriers (Hilt et al., 2007; Beevers et al., 2009; Caldwell et al., 2013). Study 2 extends these earlier reports, as neurocognitive vulnerability was evident in relation to the use of problem-focused coping among Val/Val carriers at high levels of perceived social isolation (Figure 4). Although Val/Val individuals reported greater use of problem-focused coping at low levels of social isolation, this was no longer evident with high feelings of loneliness. These data are consistent with the evidence suggesting that neurotrophins may be involved in appraisal and coping processes, and highlight that negative contextual stressors, including feelings of loneliness, may be most adverse for those with elevated neurotrophic support.

### Limitations

The present studies are subject to several limitations. As these findings are based on cross-sectional data, any directionality of variables assessed remains unclear, and so this may impact interpretations of the variables of interest. For instance, it is possible that depressive feelings may promote biased perceptions pertinent to the self-reported social isolation and self-efficacy appraisal measures. In addition, these studies made use of Caucasian undergraduate students, and so caution is advised against generalizability to other ethnic groups or circumstances. Furthermore, the samples in both studies were limited and included few individuals with the Met/Met genotype. Clearly, a larger sample would have allowed for more precise analyses to evaluate whether the differential susceptibilities evident in the present investigations may be more graded based on Val and Met allele combinations. It may also be argued that as a number of the predictors are moderately correlated, including loneliness with self-efficacy, and with emotion-focused coping, multicollinearity may have influenced the results and reduced statistical power (Tabachnick and Fidell, 2013). However, standardized predictors were used in the regression analyses, as is indicated to reduce the influence of collinearity. Moreover, the results from the moderational analyses were not found to differ when tested again with inclusion of squared predictor terms as covariates, which alleviates concern of confounding nonlinear effects associated with multicollinearity (Cortina, 1993). Considered further, given that the patterns of findings were similar across the two studies, it suggests that the results presented in the present paper are tenable, although future studies should seek to corroborate these data.

## Conclusions

With increasing evidence linking neurotrophins to appraisal and coping processes, and the data indicating that negative situational influences, including feelings of loneliness, may be especially disturbing for those with elevated neurotrophic support. In essence, the Val/Val allele variant is not necessarily always advantageous, particularly in associations involving feelings of loneliness and depression, and when other cognitive processes are considered, Met carriers may exhibit greater resilience to affective outcomes. This conclusion is in keeping with the view previously expressed in relation to serotonin (Belsky et al., 2007, 2009) and oxytocin (McQuaid et al., 2013), that “for better or worse” the Val/Val genotype for BDNF may increase sensitivity and/or responsivity to environmental stimuli, and thus influence mood state.

## Ethics statement

The study was carried out in accordance with the recommendations of the Carleton University Psychology Research Ethics Board, with written informed consent from all subjects. All subjects gave written informed consent in accordance with the Declaration of Helsinki. The study protocol was approved by the Carleton University Psychology Research Ethics Board.

## Author contributions

MB, RW, CC, and HA contributed to the inception and design of the current studies. Testing, data collection, and processing of biological samples, were performed by MB, RW, and CC. Data analyses and interpretations were performed by MB under the supervision of HA. The manuscript was drafted by MB, and HA, RW, and CC provided critical revisions. All authors approved the final version of the manuscript for submission.

### Conflict of interest statement

The authors declare that the research was conducted in the absence of any commercial or financial relationships that could be construed as a potential conflict of interest.
